# Down-regulation of LncRNA TUG1 enhances radiosensitivity in bladder cancer via suppressing HMGB1 expression

**DOI:** 10.1186/s13014-017-0802-3

**Published:** 2017-04-04

**Authors:** Huijuan Jiang, Xigang Hu, Hongzhi Zhang, Wenbo Li

**Affiliations:** Department of Radiotherapy, Huaihe Hospital of Henan University, No.1 Baobei Road, Gulou District, Kaifeng, 475000 China

**Keywords:** lncRNA, TUG1, HMGB1, Bladder cancer, Radiosensitivity

## Abstract

**Background:**

Long non-coding RNAs (lncRNAs) have been reported to regulate the sensitivity of different cancer cells to chemoradiotherapy. Aberrant expression of lncRNA Taurine-upregulated gene 1 (TUG1) has been found to be involved in the development of bladder cancer, however, its function and underlying mechanism in the radioresistance of bladder cancer remains unclear.

**Methods:**

Quantitative real-time PCR (qRT-PCR) was conducted to measure the expression of TUG1 and HMGB1 mRNA in bladder cancer tissues and cell lines. HMGB1 protein levels were tested by western blot assays. Different doses of X-ray were used for radiation treatment of bladder cancer cells. Colony survival and cell viability were detected by clonogenic assay and CCK-8 Kit, respectively. Cell apoptosis was determined by flow cytometry. A xenograft mouse model was constructed to observe the effect of TUG1 on tumor growth in vivo.

**Results:**

The levels of TUG1 and HMGB1 were remarkably increased in bladder cancer tissues and cell lines. Radiation treatment markedly elevated the expression of TUG1 and HMGB1. TUG1 knockdown inhibited cell proliferation, promoted cell apoptosis and decreased colony survival in SW780 and BIU87 cells under radiation. Moreover, TUG1 depletion suppressed the HMGB1 mRNA and protein levels. Furthermore, overexpression of HMGB1 reversed TUG1 knockdown-induced effect in bladder cancer cells. Radiation treatment dramatically reduced the tumor volume and weight in *xenograft model*, and this effect was more obvious when combined with TUG1 silencing.

**Conclusion:**

LncRNA TUG1 knockdown enhances radiosensitivity of bladder cancer by suppressing HMGB1 expression. TUG1 acts as a potential regulator of radioresistance of bladder cancer, and it may represent a promising therapeutic target for bladder cancer patients.

## Background

Bladder cancer, the ninth most common cancer worldwide, can be distributed into two clinically different groups, non-muscle invasive bladder cancer (NMIBC) and muscle invasive bladder cancer (MIBC) [1]. Although chemoradiotherapy as an adjuvant treatment of surgery was developed, it failed to universally improve outcomes due to the resistance of bladder cancers to chemoradiotherapy [2]. Therefore, further excavate the potential mechanisms of radioresistance and improve radiosensitivity in patients with bladder cancer become very urgent.

High mobility group box 1 protein (HMGB1), a chromosome-binding protein, functions as a DNA chaperone and involves in many physiological processes in the cell nucleus, including DNA repair, duplication, transcription, and nucleosome packaging [3, 4]. When translocated to the cytoplasm, HMGB1 could invoke autophagy via interacting with beclin-1 [5]. HMGB1 from the extracellular medium warns surrounding cells and immune system to urgent danger, contributing to inflammation [6]. Overexpression of HMGB1 was observed in several cancers, and played significant roles in regulation of tumor growth, metastasis, and chemotherapy and radiotherapy resistance [7–10]. HMGB1 is capable of promoting both chemoresistance and radioresistance in breast cancer cells [11, 12]. However, the function of HMGB1 in bladder cancer carcinogenesis and radioresistance remains poorly understood.

Long non-coding RNAs (lncRNAs) are non-protein-coding transcripts longer than 200 nucleotides, and exert their physiological and pathological functions through their interactions with genomic DNA, miRNAs, mRNAs and proteins [13]. Increasing evidence suggests that lncRNAs are important molecules involved not only in normal development but also in tumorigenesis [14]. Abnormal expression lncRNAs could serve as oncogenes and tumor suppressors, closely associated with tumorigenesis, metastasis, prognosis or diagnosis [15]. Moreover, accumulating documents reveal that lncRNAs are related to radiotherapy resistance of cancers [16–18]. For instance, HOTAIR expression was upregulated in tumor tissues of colorectal cancer (CRC) patients, and HOTAIR knockdown inhibited proliferation, migration and invasiveness while enhanced apoptosis and radiosensitivity of CRC cells [18]. HOTAIR expression was markedly increased in the pancreatic ductal adenocarcinoma (PDAC) cell lines and tissues, and HOTAIR silencing improved the radiosensitivity of PDAC cells via regulating the expression of Wnt inhibitory factor 1 (WIF-1) [16].

Taurine-upregulated gene 1 (TUG1) was firstly reported to be upregulated in exposure to the treatment of taurine in mouse retinal cells [19]. TUG1 has been proved to act as a tumor suppressor or oncogene in various cancers [20–22]. In addition, aberrant expression of TUG1 has been observed in bladder cancer cells. For instance, TUG1 expression was remarkably increased in high-grade MIBC tumor tissues, and TUG1 silencing suppressed proliferation and migration in high-grade MIBC [23]. TUG1 was upregulated in bladder cancer tissues and cell lines, and promoted cancer cell invasion and radioresistance through inducing epithelial-to-mesenchymal transition (EMT) [24]. However, the function and molecular mechanism of TUG1 in bladder cancer radioresistance is still largely undefined.

In the present study, we aimed to investigate the effect of TUG1 on the radiosensitivity of bladder cancer cells and its underlying molecular mechanisms.

## Methods

### Patient samples and cell lines

Thirty-nine pairs of bladder cancer tissues and matched adjacent normal tissues were obtained from Huaihe Hospital of Henan University. The Clinical characteristics of patients with bladder cancer were shown in Table 1. Written consents from all patients and approval of Huaihe Hospital Ethic Review Committees were obtained prior to the use of these clinical materials.

**Table 1:** Patient demographics and clinical characteristics

Demographic or clinical characteristic	No. of patients
	(*N* = 39)
Age, years	
Median	67
Range	56–89
Sex	
Male	27
Female	12
Primary site	
Bladder	34
Renal pelvis	5
Histology	
TCC	32
SCC/adenocarcinoma/neuroendocrine	7
Stage^a^	
0cis/0a/1	5
2/3	18
4	16

*Abbreviations*: *TCC* Transitional cell carcinoma, *SCC* Squamous cell carcinoma

^a^Stages are defined as follows: 0cis = carcinoma in situ; 0a = noninvasive papillary carcinoma; 1 = tumor invades subepithelial connective tissue; 2/3 = invasive tumor, muscle/perivesicular tissue; and 4 = metastatic disease

Normal bladder epithelial cell line (HCV-29) and human bladder cancer cell lines (SW780, HT1376, BIU87 and T24) were obtained from the American Type Culture Collection (ATCC) and cultured at 37 °C in humidified 5% CO2. All cell lines were cultured in RPMI 1640 (Gibco, Grand Island, NY, USA) supplemented with 10% fetal bovine serum, 100 U/mL penicillin, and 100 mg/mL streptomycin.

### Quantitative real-time PCR (qRT-PCR)

TRIzol reagent (Invitrogen, Carlsbad, CA, USA) was used to extract the total RNA from bladder cancer tissues and cell lines. cDNAs were synthesized by reverse transcription using M-MLV reverse transcriptase (Invitrogen). Oligo (dT18) RT primer was used for the reverse transcription of HMGB1 mRNA and lncRNA TUG1. qRT-PCR analysis was performed using SYBR Premix Ex Taq II (TaKaRa, Dalian, China) with an ABI 7500 Real-Time PCR system (Applied Biosystems, Foster City, CA, USA). β-actin was used as endogenous controls. The gene specific primers were as follows: LncRNA TUG1 (forward: 5’-CTGAAGAAAGGCAACATC-3’; reverse: 5’-GTAGGCTACTACAGGATTTG-3’); HMGB1 (forward: 5’-ACATCCAAAATCTTGATCAGTTA-3’; reverse: 5’-AGGACAGACTTTCAAAATGTTT-3’); β-actin (forward: 5’-TGAGAGGGAAATCGTGCGTGAC-3’; reverse: 5’-AAGAAGGAAGGCTGGAAAAGAG-3’).

### Western blot analysis

Protein concentrations in the whole-cell lysates were detected by using a BCA Protein Assay Kit (Pierce, Rockford, IL, USA). Then, protein samples were separated by 10% SDS-PAGE and transferred to polyvinylidene fluoride (PVDF) (Amersham Pharmacia, Little Chalfont, UK). The immunoblots were conducted by incubation with specific antibodies for HMGB1 (Cat. No: sc-56698) and β-actin (Cat. No: sc-47778). All antibodies were purchased from Santa Cruz Technology (Santa Cruz, CA, USA). Image Quant software (Molecular Dynamics, Sunnyvale, CA, USA) was used to analyze the protein bands.

### Cell transfection

Three TUG1 siRNAs (si-TUG1#1, si-TUG1#2 and si-TUG1#3) and the scramble negative controls were synthesized and purchased from GenePharma (Shanghai, China). The sequences of the three designed TUG1 siRNAs were as follows: si-TUG1 1#, CAGUCCUGGUGAUUUAGACAGUCUU; si-TUG1 2#, CCCAGAAGUUGUAAGUUCACCUUGA; si-TUG1 3#, CAGCUGUUACCAUUCAACUUCUUAA. Preliminary experiments were performed in both SW780 and BIU87 cells transfected with 100 nM different si-TUG1 to identify the most efficient siRNA sequences. To amplify HMGB1, SW780 and BIU87 cells were transfected with 5 μg pcDNA-HMGB1 (GenePharma). All transfection was performed using Lipofectamine 2000 reagent (Invitrogen) according to the manufacturer’s instructions.

### Clonogenic assay

Transfected SW780 and BIU87 cells were treated with a range of radiation doses (0, 2, 4, 6 and 8 Gy). When there was visible colony by naked eye, cells were fixed with methanol and stained with 1% crystal violet solution (Sigma, St. Louis, MO, USA) for 20 min. Colonies with more than 50 cells were then counted under an inverted microscope. Sigmaplot software (Systat, San Jose, CA, USA) was used to fit the data to a linear-quadratic model.

### Flow cytometry

The Annexin V-FITC Apoptosis Detection Kit (Sigma) was used to detect cell apoptosis in accordance with the manufacturer’s instructions. Briefly, cells were harvested and stained in the dark for 15 min at room temperature using 5 μL Annexin V-FITC and 10 μL propidium iodide (PI). Then the BD FACS Diva software V6.1.3 (BD Biosciences, San Jose, CA, USA) was used to analyze the flow cytometry data.

### Cell viability assays

SW780 and BIU87 cells transfected with si-TUG1 or si-TUG1 and pcDNA-HMGB1 were seeded in a 96-well plate for 24 h, and then cells were exposed to 2 Gy radiation. At 0, 24, 48, 72 and 96 h after radiotherapy, cell viability was determined by the Cell Counting Kit-8 Kit (Dojindo, Kumamoto, Japan) in accordance with the manufacturer’s protocol.

### Xenograft mouse model

Total 2 × 10^6^ non-transfected or transfected (si-NC or si-TUG1) SW780 cells were subcutaneously injected into the back of four- to six-week-old male athymic nude mice (*n* = 6 per group). To evaluate tumor radioresistance in vivo, the mice were given radiation (2 Gy) for 5 consecutive days when the tumors reached an average volume of approximately 100 mm^3^. Tumor volume was measured with slide calipers every three days after the first radiotherapy according to the formula: volume = 1/2 × length × width^2^. Three weeks later, mice were sacrificed to remove and weigh tumors. All animal procedures were performed with the approval of the Local Medical Experimental Animal Care Commission.

### Statistical analysis

Statistical analysis was performed using SPSS 19.0 software (SPSS, Chicago, IL, USA). The data were expressed as means ± SD. The difference between groups was evaluated by the Student’s *t*-test or one-way ANOVA. *P* < 0.05 indicated a statistical significance.

## Results

### The level of TUG1 is positively correlated with HMGB1 expression in bladder cancer tissues

**Fig. 1:**
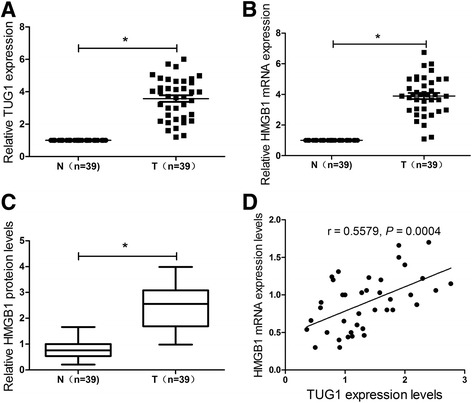
Expression of TUG1 and HMGB1 in bladder cancer tissues. **a** and **b** The expression of TUG1 and HMGB1 mRNA was measured by qRT-PCR analysis in bladder cancer tissues (*n* = 39) or adjacent non-cancer tissues (*n* = 39). **c** The protein level of HMGB1 was detected by western blot in bladder cancer tissues (*n* = 39) or adjacent non-cancer tissues (*n* = 39). **d** Correlation analysis of TUG1 and HMGB1 expression in bladder cancer tissues. *P < 0.05 vs. control

The expression of TUG1 and HMGB1 mRNA was measured by qRT-PCR and the HMGB1 protein level was determined by western blot in bladder cancer tissues and adjacent non-cancer tissues, respectively. The expression level of TUG1 (Fig. 1a) and HMGB1 mRNA (Fig. 1b) and protein (Fig. 1c) was about twice higher in bladder cancer tissues than that in adjacent normal tissues. Moreover, the level of TUG1 is positively related to HMGB1 expression in bladder cancer tissues (Fig. 1d). These results suggested that upregulation of TUG1 and HMGB1 may be involved in the pathogenesis of bladder cancer.

### Radiation treatment increases the TUG1 expression and HMGB1 protein level in bladder cancer cell lines

**Fig. 2:**
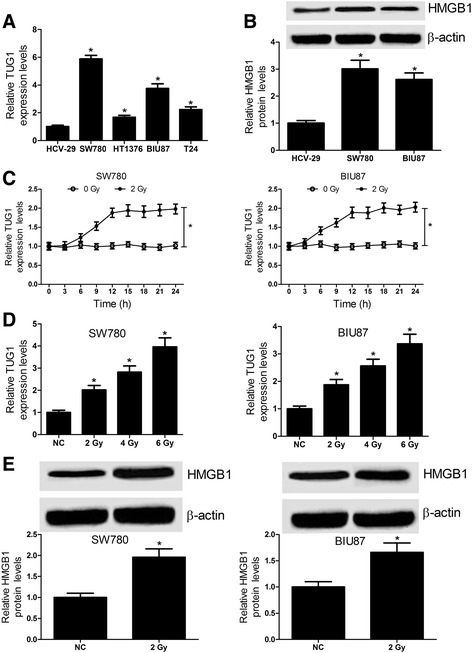
Radiation promotes the TUG1 and HMGB1 expression in bladder cancer cell lines. **a** qRT-PCR analysis was conducted to detect the TUG1 expression in bladder cancer cell lines SW780, HT1376, BIU87 and T24 and normal bladder epithelial cell line HCV-29. **b** The protein level of HMGB1 was determined by western blot in SW780 and BIU87 cells. **c** TUG1 expression was detected in SW780 and BIU87 cells every 3 h after 0 or 2 Gy of ionizing radiation treatment. **d** TUG1 expression was measured in SW780 and BIU87 cells after different doses (0, 2, 4, 6 Gy) of ionizing radiation treatment for 24 h. **e** The HMGB1 protein level was upregulated after SW780 and BIU87 cells treated with 2 Gy of ionizing radiation for 24 h. *P < 0.05 vs. NC

To test the expression change of TUG1 and HMGB1 in bladder cancer cell lines, qRT-PCR and western blot were performed, respectively. TUG1 was significantly increased in bladder cancer cell lines (SW780, HT1376, BIU87 and T24) compared with normal bladder epithelial cell line HCV-29 (Fig. 2a), especially in SW780 and BIU87 cells. Similarly, the HMGB1 protein level was dramatically elevated in SW780 and BIU87 cells (Fig. 2b). To further determine the effect of radiation treatment on TUG1 and HMGB1 expression, SW780 and BIU87 cells were treated with 2 Gy of radiation. qRT-PCR analysis manifested that the TUG1 expression in SW780 and BIU87 cells (Fig. 2c and d) was dramatically increased after radiation treatment in time- and dose-dependent manners. Western blot results indicated that the HMGB1 protein level was increased after radiation exposure in both SW780 and BIU87 cells (Fig. 2e). Taken together, all these results revealed that radiation treatment promotes the TUG1 and HMGB1 expression in bladder cancer cell lines.

### TUG1 knockdown enhances radiosensitivity of bladder cancer cell lines

**Fig. 3:**
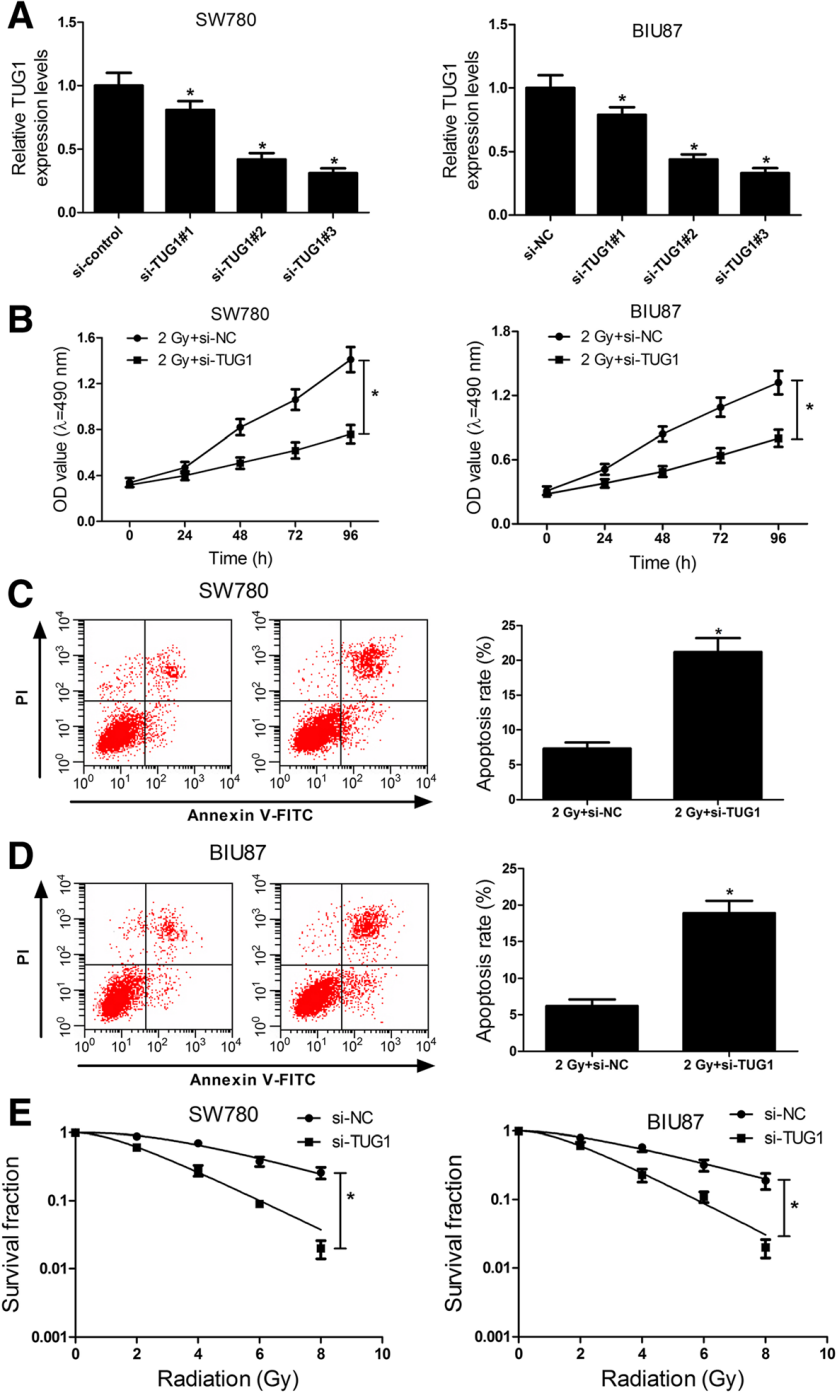
TUG1 silencing enhances radiosensitivity of bladder cancer cell lines. **a** qRT-PCR analysis of the relative TUG1 expression in SW780 and BIU87 cells transfected withTUG1-specific si-RNAs or control si-RNA. SW780 and BIU87 cells were transfected with si-NC or si-TUG1 and cultured for 24 h followed by ionizing radiation. **b** The cell viability of transfected SW780 and BIU87 cells at 0, 24, 48, 72 and 96 h afterionizing radiation. **c** and **d** Cell apoptosis was determined in transfected SW780 and BIU87 cells at 24 h after 2 Gy radiotherapy. **e** Transfected SW780 and BIU87 cells were subjected to 0, 2, 4, 6 and 8 Gy of irradiation. After 2 weeks, the colony survival fractions were measured. *P < 0.05 vs. si-NC

Considering that radiation treatment could enhance the TUG1 expression (Fig. 2c), we assumed that TUG1 may be related to the radiation resistance in bladder cancer. The colony survival assay is considered as a canonical standard to determine radiosensitivity [25]. Moreover, the anti-apoptosis activities of cancer cells are closely related to radioresistance. To confirm whether TUG1 could affect the radiosensitivity of bladder cancer cells, loss-of-function assay was performed by transfecting si-TUG1 (siRNAs specific to TUG1) into SW780 and BIU87 cells. Among the designed three siRNAs (si-TUG1#1, si-TUG1#2 and si-TUG1#3), si-TUG1#3 was validated to possess the highest knockdown efficiency in SW780 and BIU87 cells (Fig. 3a) and was subsequently used for further experiments. SW780 and BIU87 were transfected with si-NC or si-TUG1 and incubated for 24 h, then the cells were exposed to 2 Gy of irradiation. At indicated time points, cells were used for proliferation and apoptosis analysis. CCK-8 assay manifested that TUG1 knockdown markedly impaired the cell growth of SW780 and BIU87 cells compared with control group (Fig. 3b). Flow cytometry analysis revealed that the apoptosis rates of SW780 and BIU87 cells were prominently enhanced by TUG1 silencing (Fig. 3c and d). Moreover, clonogenic assay indicated that TUG1 downregulation significantly lowered the survival fractions in SW780 and BIU87 cells (Fig. 3e) compared with controls. Collectively, these data demonstrated that TUG1 knockdown enhances radiosensitivity of bladder cancer cells SW780 and BIU87.

### TUG1 knockdown inhibits the HMGB1 expression in bladder cancer cell lines

**Fig. 4:**
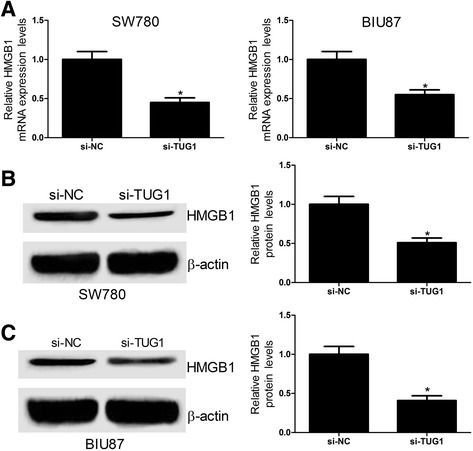
TUG1 downregulation inhibits the HMGB1 expression in bladder cancer cell lines. **a** The expression of HMGB1 mRNA was detected by qRT-PCR analysis at 24 h after SW780 and BIU87 cells were transfected with si-NC or si-TUG1. **b** and **c** The protein level of HMGB1 was examined at 24 h after SW780 and BIU87 cells were transfected with si-NC or si-TUG1. *P < 0.05 vs. si-NC

Considering the positive correlation between TUG1 and HMGB2 expression in bladder cancer tissues and cells, as well as their consistent change under radiation treatment, we assumed that TUG1 may affect the radiosensitivity of bladder cancer cells via regulating the expression of HMGB2. To verify this hypothesis, the effect of TUG1 knockdown on HMGB1 expression in bladder cancer cells was first assessed., qRT-PCR and western blot showed that the mRNA (Fig. 4a) and protein (Fig. 4b and c) levels of HMGB1 were dramatically downregulated in si-TUG1-transfected SW780 and BIU87 cells. All these results illustrated that silencing of TUG1 inhibited the HMGB1 expression in bladder cancer cell lines.

### Restoration of HMGB1 expression relieves the enhanced radiosensitivity of bladder cancer cells caused by TUG1

**Fig. 5:**
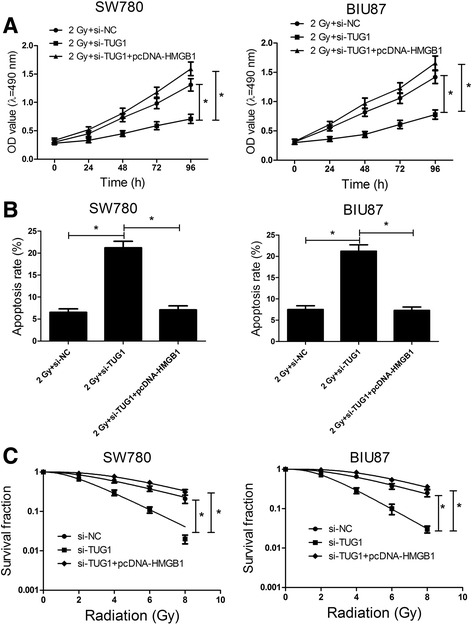
Restoration of HMGB1 expression reversed the increased radiosensitivity of bladder cancer cells induced by TUG1 knockdown. SW780 and BIU87 cells were transfected with si-TUG1 or co-transfected with si-TUG1 and pcDNA-HMGB1. At 24 h post transfection, cells were exposed to X-ray for radiation. **a** Cell proliferation was detected using CCK-8 Kit at the indicated times after 2 Gy of irradiation treatment. **b** Cell apoptosis was tested by flow cytometry at 24 h after radiotherapy. **c** The clonogenic survival curve was established on day 14 after transfected cells received indicated doses of radiation. *P < 0.05 vs. si-NC

To further investigate whether TUG1 knockdown enhanced radiosensitivity of bladder cancer cells via inhibiting HMGB1, SW780 and BIU87 cells transfected with si-TUG1 or combination of si-TUG1 and pcDNA-HMGB1 were treated with 2 Gy radiation. Restored expression of HMGB1 by pcDNA-HMGB1 reversed the inhibited cell viability (Fig. 5a), increased apoptosis (Fig. 5b) and reduced survival fractions (Fig. 5c) induced by the depletion of TUG1 in SW780 and BIU87 cells. To sum up, these results revealed that knockdown of TUG1 enhances radiosensitivity of bladder cancer cells by downregulating HMGB1 expression.

### TUG1 knockdown sensitized bladder cancer cells to irradiation in vivo

**Fig. 6:**
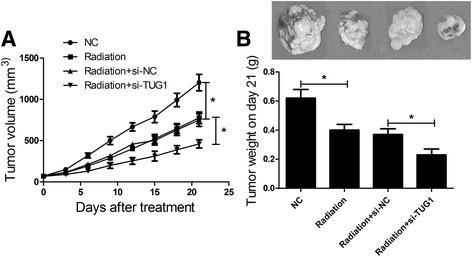
TUG1 knockdown enhances radiosensitivity of bladder cancer cells in vivo. SW780 cells were subcutaneously injected into the back of male athymic nude mice (*n* = 6 per group). All mice were given radiation (2 Gy) for 5 consecutive days when the tumors reached an average volume of about 100 mm^3^. **a** Tumor volume was measured every 3 days after the first radiotherapy. **b** Mice were euthanatized to resect and weigh tumors after 3 weeks. *P < 0.05 vs. NC or si-NC

To determine the effect of TUG1 knockdown on the radiosensitivity of the bladder cancer mouse model, non-transfected or transfected SW780 cells were injected subcutaneously into nude mice. When the xenografts reached a certain volume, the mice received a 2 Gy irradiation treatment for successive 5 days. The results demonstrated that radiation treatment significantly inhibited the tumor growth, including the average volume (Fig. 6a) and weight (Fig. 6b), and this effect was more evident when combined with TUG1 knockdown. Together, TUG1 depletion improved the radiosensitivity of bladder cancer cells in vivo.

## Discussion

In the present study, we explored function and underlying mechanisms of lncRNA TUG1 in the radiosensitivity of bladder cancer. Our study declared that TUG1 expression was markedly upregulated in bladder cancer tissues and cell lines. Moreover, TUG1 knockdown enhanced the radiosensitivity of bladder cancer cell lines, evidenced by the reduced cell viability, the increased cell apoptosis and the inhibited colony survival fractions. Furthermore, our findings suggest that TUG1 knockdown enhances radiosensitivity of bladder cancer cells in vivo and in vitro by suppressing the expression of HMGB1.

LncRNA TUG1 was overexpressed in several kinds of cancer tissues such as esophageal carcinoma [20], osteosarcoma [22] and hepatocellular carcinoma [26]. However, TUG1 was downregulated in non-small cell lung cancer and glioma [21, 27]. All these studies revealed that TUG1 could function as an oncogene or tumor suppressor in different cancers. In the present study, we found that TUG1 expression was increased in bladder cancer tissues and cell lines, which was consistent with the data of previous studies [22–24]. Moreover, radiation increased TUG1 expression, which implied that TUG1 may be associated with the radioresistance in bladder cancer. TUG1 knockdown inhibited cell proliferation and colony survival fractions, as well as induced cell apoptosis, which was supported by a previous finding that TUG1 inhibition upregulated the expression of the apoptosis-inducing factors such as AIF, AIP1, NIP3, and NIPOR, and suppressed cell growth and promoted apoptosis [28]. Additionally, radiation significantly blocked the tumor growth of mouse model, and the inhibitory effect was enhanced when combined with TUG1 knockdown. All these findings demonstrate that TUG1 knockdown contributes to the increase of radiosensitivity of bladder cancer cells in vivo and in vitro. It was reported that TUG1 promoted radioresistance of bladder cancer [24], which agrees with our findings.

HMGB1 was found to be overexpressed and could regulate tumor growth, metastasis and survival in cancers, including bladder cancer [29]. HMGB1 is also related to the chemoresistance and radioresistance in various cancers [30–32]. For instance, HMGB1 could mediate the chemotherapy resistance in breast cancer [11]. HMGB1 knockdown could promote the radiosensitivity of breast cancer cells through breaking telomere homeostasis and inhibiting the repair of DNA damage [12]. Moreover, HMGB1 knockdown markedly sensitize the bladder cancer cells to radiotherapy [33]. However, how it is regulated in radiosensitivity of bladder cancer is not quite clear. This study presented a positive correlation between TUG1 and HMGB1 expression, and their changes were consistant in response to irradiation treatment. Therefore, we explored whether TUG1 regulated the radiosensitivity of bladder cancer through modulating HMGB1 expression. In consistent with our surmise, our findings reveal that IncRNA TUG1 knockdown enhances radiosensitivity of bladder cancer through suppressing HMGB1 expression. A previous study found that TUG1 promoted cancer cell invasion and radioresistance through inducing epithelial-to-mesenchymal transition (EMT) in bladder cancer [24]. In addition, a previous document revealed that elevated TUG1 expression was closely correlated with chemotherapy resistance of esophageal squamous cell carcinoma [28]. Therefore, TUG1 may be involved in the chemotherapy and radiotherapy resistance in cancers, providing a theoretical foundation for the clinical application of TUG1 in patients with radio- and chemo-resistant cancer.

## Conclusions

In summary, TUG1 expression was upregulated in bladder cancer tissues and cell lines. Downregulation of TUG1 enhanced the radiosensitivity of bladder cancer cells via inhibiting HMGB1 expression. Our findings suggest that TUG1 could be a promising therapeutic target for bladder cancer and combination treatment of TUG1 knockdown and radiation may be a better strategy for the patients with radioresistant bladder cancer.

## Abbreviations

CRC: Colorectal cancer
EMT: Epithelial-to-mesenchymal transition
HMGB1: High mobility group box 1 protein
LncRNAs: Long non-coding RNAs
MIBC: Muscle invasive bladder cancer
NMIBC: Non-muscle invasive bladder cancer
PDAC: Pancreatic ductal adenocarcinoma
qRT-PCR: quantitative real-time PCR
TUG1: Taurine-upregulated gene 1
TUG1: Taurine-upregulated gene 1
WIF-1: Wnt inhibitory factor 1
